# Enteric pathogens and associated risk factors among under-five children with and without diarrhea in Wegera District, Northwestern Ethiopia

**DOI:** 10.11604/pamj.2018.29.72.13973

**Published:** 2018-01-24

**Authors:** Hailemariam Feleke, Girmay Medhin, Almaz Abebe, Birhan Beyene, Helmut Kloos, Daniel Asrat

**Affiliations:** 1 Ethiopian Institute of Water Resources, Addis Ababa University, Ethiopia; 2 Department of Biology, Assosa University, Ethiopia; 3 Aklilu Lemma Institute of Pathobiology, Addis Ababa University, Ethiopia; 4 Ethiopian Public Health Institute, Ethiopia; 5 Medical Center, University of California, San Francisco, USA; 6 Faculty of Medicine, Addis Ababa University, Ethiopia

**Keywords:** Acute diarrhea, under-five children, enteric pathogens, rotavirus vaccination, sociodemographic and environmental factors, Ethiopia

## Abstract

**Introduction:**

Childhood diarrhea is highly prevalent in slums in developing countries, but it remains understudied. The objectives of this study were to explore the prevalence of *Giardia*, rotavirus and bacterial enteropathogens among diarrheic and non-diarrheic children and investigate socio-environmental determinants of diarrhea in two Ethiopian towns.

**Methods:**

A cross-sectional study was conducted from June to October 2016. Prevalence of childhood diarrhea was established using information gathered during interviews with mothers/guardians. Saline wet mounts of fresh stool samples were used to test for the presence of *Giardia*. Stool samples were cultured on MacConkey agar and suspected colonies were characterized using biochemical tests. Susceptibility testing was done by the disk diffusion method. ELISA was used to screen for rotavirus.

**Results:**

A total of 225 children were included in this study. Four enteropathogens (*Giardia*, rotavirus, *Shigella* and *Salmonella*) were identified from 31% (35/112) diarrheic and 12% (14/113) from non-diarrheic children (*p* < 0.001). The prevalence of rotavirus infection was 18.0% among diarrheic children and 3.3% among non-diarrheic children unvaccinated against rotavirus (*p* < 0.01). The prevalence of *Giardia* was 21.0% among diarrheic and 8.0% among non-diarrheic children (*p* < 0.01). Diarrheic children had significantly higher rates of bloody stool (*p* < 0.02), vomiting, fever and breastfeeding for children beyond 23 months of age (*p* < 0.001). Giardia and rotavirus were identified in more diarrheic than non-diarrheic children.

**Conclusion:**

The high prevalence of *Giardia* and rotavirus in the study area indicates the need for coordinated healthcare activities in the two communities. Vaccination against rotavirus infections and educational interventions are recommended.

## Introduction

Diarrhea due to infectious agents is a major global health problem. An estimated 4 billion cases contract diarrhea each year and more than 1.5 million children die every year due to diarrhea [1]. The situation is critical in developing countries due to inadequate potable water supplies; limited sanitation and poor hygiene practices. Throughout Africa, diarrhea is a leading killer of children [2] and about 14% of the deaths among children in Ethiopia are due to diarrhea [2]. Young children are particularly vulnerable to acute diarrhea, which is generally defined as having at least three loose stools within a 24hr period [3]. Sociocultural factors, poor quality drinking water, lack of formal education and low hygiene levels are known risk factors for diarrhea [4,5]. Socio-demographic, behavioral and environmental characteristics are common risk factors for diarrhea [6]. Diarrhea caused by enteropathogens is a serious health burden in developing countries [7]. Various species of *Giardia*, rotavirus, *Shigella* and *Salmonella* are among the most common diarrhea-causing pathogens transmitted through poor quality water and unhygienic conditions [8–13]. Goitom et al. 2017, in an article in press showed that a decrease in rotavirus positivity was inversely related to an increase in rotavirus vaccine coverage, indicating the impact of rotavirus vaccines [14]. However, rotavirus vaccination coverage for young children in Ethiopia is 47% [15]. Infectious diarrhea due to viral, bacterial, protozoal or helminthic organisms in children can be minimized by providing adequate supplies of safe drinking water and practicing better hygiene [16,17]. Though infectious agents of diarrhea have been widely studied [18], few studies have been carried out on these pathogens, particularly rotavirus and bacterial enteropathogens [19,20] and associated risk factors of acute diarrhea in Ethiopia. The frequency with which children grow up in deficient housing is a neglected but important aspect of health inequality [21] and its effect on child health is often more pronounced in urban than in rural settings [21]. The aim of this study was to determine the prevalence of *Giardia*, rotavirus and bacterial enteropathogens in under-five children and to examine factors associated with acute diarrhea in two towns in Wegera District, Ethiopia.

## Methods

Ethics approval and consent to participate

Ethical clearance was obtained from the Ethiopian Public Health Institute as well as a study permit from the Wegera District Health Bureau prior to the conduct of the study. Written consent was obtained from respondents. Children who were positive for enteric pathogens were treated in health centers based on the national treatment protocol [22].

### Study area

**Figure 1:**
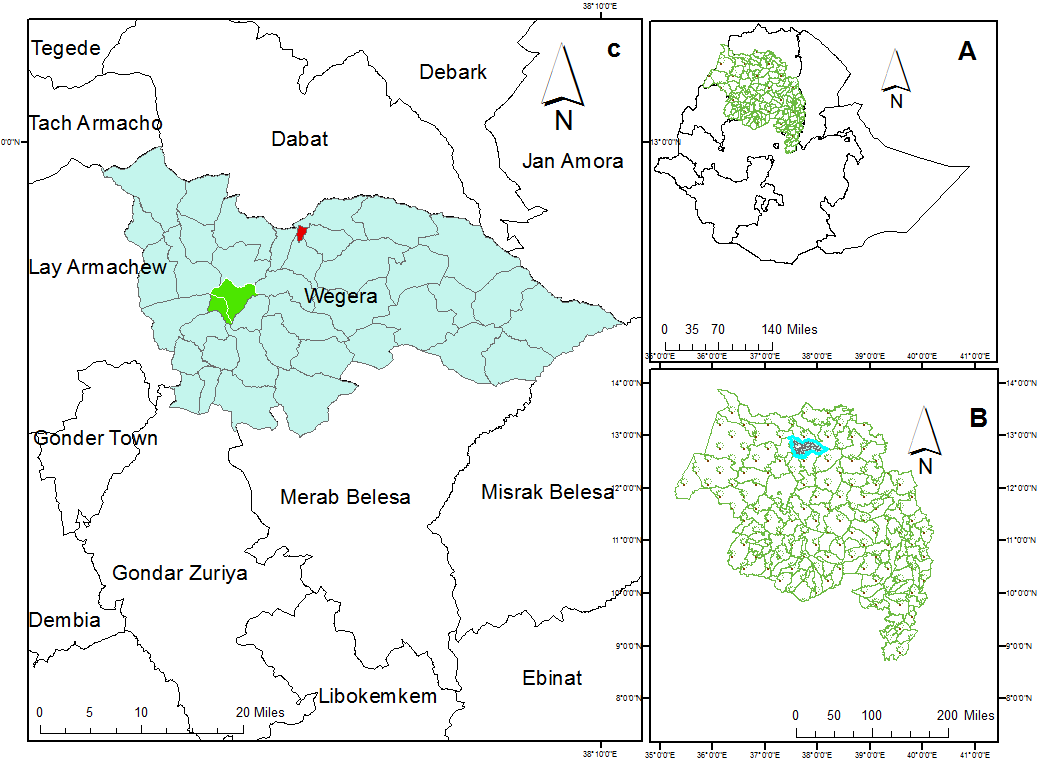
Map of Amhara Region (A), Wegera District (B), Ambagiorgis and Gedebge towns (red color) (C)

The study was conducted in Wegera District in Amhara Regional State in the northern Ethiopian highlands. Wegera District had a total population of 256,608 during the 2007 national census. The towns of Ambagiorgis and Gedebge, where the study was carried out, had 22,000 and 8,000 residents, respectively (Figure 1).

### Study design

A cross-sectional study design was used with data collection carried out from June to October 2016. One hundred forty-six children (74 diarrheic and 72 non-diarrheic) in Ambagiorgis and 79 children (38 diarrheic and 41 non-diarrheic) in Gedebge were enrolled. The prevalence of diarrhea was determined from interviews with parents/guardians during house-to-house visits. The source population all came from households in Ambagiorgis and Gedebge towns having at least one under-five child. Nine hundred households from Ambagiorgis and 325 households from Gedebge towns, each having at least one under-five child, were fully registered and participants randomly selected. A systematic random sampling technique was used to identify the participating children. During interviews, parents/guardians of children were asked to visit the health center for diagnosis. Stool samples were collected for clinical diagnosis of the children in the health center.

### Sample size estimation

The sample size was calculated using “Epi-info 7”. Based on similar studies, the researchers assumed the minimal prevalence of breastfed children up to 6 month of age to be 78% among diarrheic and 92% among non-diarrheic under-five children [23]. The alpha error was set to 5% and the power of the study was set at 80%. This gave a sample size of 112 diarrheic and 113 non-diarrheic children, considering a non-response of 0.9% among children with acute diarrhea.

### Data collection

Using the pre-tested and structured questionnaire parents/guardians were interviewed by data collectors in their houses on behalf of their children. The information they provided was validated by direct observation and by birth and vaccine cards. The information on child age and vaccination was validated by observing birth and vaccine cards, respectively. Bloody stool and vomiting were confirmed by direct observation for 10% of the children (20 diarrheic and 2 non-diarrheic) and fever was validated by thermometer in the axilla of the child. Types of water storage, refuse disposal, and latrine facilities were verified by direct observation. All the participating mothers/guardians responded in the interview and stool samples were provided by 98% and 99% of the diarrheic and non-diarrheic children, respectively.

### Stool sample collection, transportation and analysis

Six health workers were recruited and trained by the principal investigator for two days on sample collection. Mothers were asked to take their children to the health center. From each child, 5-10ml of freshly passed single stool samples were collected using three different 25ml plastic stool cups. To clinically identify diarrheic and non-diarrheic children, information from the parents/guardians on diarrhea occurrence among the children was validated by clinical diagnosis of the children, and the stool samples were collected from 98% of diarrheic and 99% of non-diarrheic children. The first stool sample was used to examine for *Giardia* at the health center. Direct wet mounts with normal saline (0.85% NaCl) solution were prepared and observed under a light microscope at 10X and 40X magnification [24]. The second sample was transferred into two tubes of 1.5ml sterile Eppendorf and kept at -80°C for rotavirus analysis at the Ethiopian Public Health Institute in Addis Ababa. The third sample was transported to the Gondar Hospital laboratory in Cary-Blair transport media for *Shigella* and *Salmonella* tests.

### Rotavirus screening

Rotavirus screening was done in the Virology Laboratory of the Ethiopian Public Health Institute using an ELISA kit (Dakoppat, Copenhagen, Denmark). A 10% stool specimen suspension was prepared, and to each 100μl of the liquid fecal specimen, 1000μl of sample diluent (specific buffered saline) was added. This mixture was homogenized in a vortex and the supernatant was segregated after allowing it to settle for 10 minutes. The test was carried out following the manufacturer’s procedures and controls. The absorbance was read using spectrophotometer (Labsystems Multiskan Ms) at 450nm and cut-off value was calculated to be 0.250 (cut off value: Negative control + 0.20, where Positive control = 1.007 and Negative control = 0.050). Results above the cut-off value were considered to be positive [25].

### Bacterial isolation

Specimens were cultured on MacConkey and Salmonella-Shigella agar (DIFCO) plates and were incubated at 37°C for 24 hours. Non-lactose fermenters were subcultured onto deoxycholate and xylose lysine deoxycholate (oxoid) agar at 37°C for 24 hours. *Salmonella* and *Shigella* isolates were identified by their growth characteristics on deoxycholate and xylose lysine deoxycholate agar. Suspected colonies were further characterized using biochemical tests to identify *Shigella* and *Salmonella* [26].

### Antimicrobial susceptibility

Susceptibility testing was carried out by standardized agar disk diffusion methods on Mueller Hinton agar (DIFCO, Voigt Global Distribution Inc, USA) by incubating at 37°C for 16-18hrs. Antibiotic disks were used with the following concentrations: ampicillin, 10μg; tetracycline, 30μg; chloramphenicol, 30μg; amoxicillin, 2μg; cotrimoxazole, 25μg; ceftriaxone, 30μg, and gentamycin, 10μg. Broth turbidity was made to match with 0.5 McFarland standards. Resistance and sensitivity were interpreted using the guidelines of the Clinical Laboratory Standard Institute [27].

### Quality control

The questionnaire was translated from English to Amharic and back to English with the help of a language professional. Health extension workers and lab technicians (male and female) conducted interviews and stool sample collections at homes and the health center, respectively. Prior to actual data collection, a pre-test was made on 5% of the study population in a similar setting in Wegera District. The principal investigator and supervisors supervised the data collection process. Throughout the data collection period, completeness and consistency of the data were checked carefully. Quality control for transport and culture media was done using an *Escherichia coli* strain (ATCC 25922), which was sensitive to all the tested drugs.

### Operational definitions

*Acute diarrhea* is an abnormally frequent discharge of semisolid or fluid fecal matter from the bowel, lasting fewer than 14 days [**28**]. *Diarrheic children* refers to participating children who had acute diarrhea during the two weeks prior to the survey. *Non-diarrheic children* denotes participating children free of acute diarrhea during the two weeks prior to the survey. *Water* refers to drinking water.

### Data management and analysis

Data were double entered in EPIDATA Version 3.1 and analysis was performed using STATA 14 software. Categorical variables were summarized using percentages. The association between diarrheic or non-diarrheic status of children and 11 independent variables (6 demographic and clinical variables and 5 environmental variables), as well as the association between diarrheic status and the four enteropathogens, were tested using a chi square test at the 0.05 level of significance.

## Results

### Sociodemographic and clinical characteristics

Background characteristics of the study participants are summarized in Table 1. The proportions of male and female children with and without acute diarrhea were similar; 39% (44/112) of males and 61% (68/112) of females were diarrheic and 45% (51/113) of males and 55% (62/113) of females were non-diarrheic (p>0.05). The stool was bloody in 61% (68/112) of diarrheic participants and 8.9% (10/113) of non-diarrheic children (p < 0.02); 86.6% (97/112) of diarrheic and 15.0% (17/113) of non-diarrheic children exhibited vomiting (p < 0.001). Fever was detected in 92.9% (104/112) of diarrheic children and 32.7% (37/113) of non-diarrheic children (p < 0.001). Among children above 23 months of age, breastfeeding was practiced by 26.8% (30/112) of the mothers/guardians of diarrheic participants and by 72.6% (82/113) of the mothers/guardians of non-diarrheic children (*p* < 0.001) (Table 1).

**Table 1:** Demographic and clinical characteristics of children under five years of age in Ambagiorgis and Gedebge

Characteristics	Diarrheic (n = 112) (%)	Non-diarrheic (n = 113) (%)	Overall (n = 225) (%)	X^2^	P value
**Sex**					
Female	44 (39.3)	51 (45.1)	95 (42.2)	0.79	0.375
Male	68 (60.7)	62 (54.9)	130 (57.8)		
**Bloody stool**					
No	44 (39.3)	103 (91.2)	147 (65.3)	66.81	0.001
Yes	68 (60.7)	10 (8.9)	78 (34.7)		
**Vomiting**					
No	15 (13.4)	96 (85.0)	111 (49.3)	115.25	0.001
Yes	97 (86.6)	17 (15.0)	114 (50.7)		
**Fever**					
No	8 (7.1)	76 (67.3)	84 (37.3)	86.88	0.001
Yes	104 (92.9)	37 (32.7)	141 (62.7)		
**Breastfeeding frequency by age group (month)**					
< 6	6 (5.4)	3 (2.7)	9 (4.0)
[6,12)	23 (20.5)	14 (12.4)	37 (16.4)	50.03	0.001
[13,24)	51 (45.5)	16 (14.2)	67 (29.8)		
≥ 24	30 (26.8)	82 (72.6)	112 (49.8)		
**Rotavirus Vaccination**				
No	36 (32.1)	44 (38.9)	80 (35.6)	1.13	0.287
Yes	76 (67.9)	69 (61.1)	145 (64.4)		

### Environmental characteristics

Among diarrheic children, 66.1% (74/112) were recruited from Ambagiorgis and the remaining 33.9% (38/112) were recruited from Gedebge towns. Similarly, among non-diarrheic children, 63.7% (72/113) were recruited from Ambagiorgis and 36.3% (41/113) were recruited from Gedebge Town (*p* > 0.05). The sources of drinking water were springs for 12.5 % (14/112) of diarrheic and 3.5% (4/113) of non-diarrheic children, and tap water for 87.5% (98/112) of diarrheic and 96.5% (109/113) of non-diarrheic children (*p* < 0.013). Wide-mouthed water storage containers; improper refuse disposal and lack of latrine facilities were observed more frequently in households of diarrheic children (*p* < 0.001) (Table 2).

**Table 2:** Environmental characteristics of children under five years of age in Ambagiorgis and Gedebge

Characteristics	Diarrheic (n = 112), (%)	Non-diarrheic (n = 113), (%)	Overall (n = 225), (%)	X^2^	P value
**Site**					
Ambagiorgis	74 (66.1)	72 (63.7)	146 (64.9)	0.14	0.711
Gedebge	38 (33.9)	41 (36.3)	79 (35.1)		
**Source of water**					
Spring water	14 (12.5)	4 (3.5)	18 (8.0)	6.14	0.013
Tap water	98 (87.5)	109 (96.5)	207 (92.0)		
**Storage container**					
Narrow mouth Wide	42 (37.5)	106 (93.8)	148 (65.8)	79.22	0.001
Mouth	70 (62.5)	7 (6.2)	77 (34.2)		
**Refuse disposal**			
Private pit	5 (4.5)	4 (3.5)	9 (4.0)	84.5	0.001
Communal pit	11 (9.8)	19 (16.8)	30 (13.3)		
Composting	31 (27.7)	10 (8.9)	41 (18.2)		
Burning	8 (7.1)	66 (58.4)	74 (32.9)		
Open field	57 (50.9)	14 (12.4)	71 (31.6)		
**Latrine facility**			
No	56 (0.5)	15 (13.3)	71 (31.6)	35.13	0.001
Yes	56 (0.5)	98 (86.5)	154 (68.4)		

### Infectious agent characteristics

**Figure 2:**
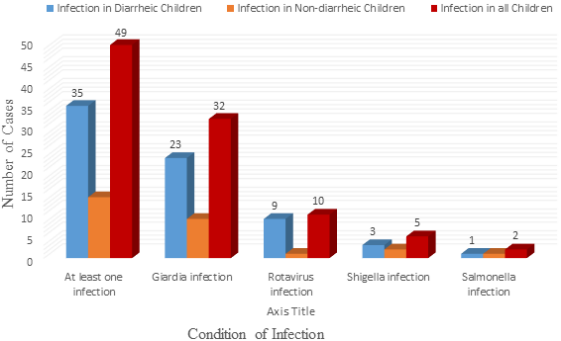
The infectious agents of diarrhea isolated from children with and without diarrhea in Ambagiorgis and Gedebge

Summary results presented in Table 3 show that 31.3% of diarrheic and 12.4% of non-diarrheic children were positive for at least one of the four infectious agents (*p* < 0.001). In particular, 20.5% (23/112) of diarrheic and 8.0% (9/113) of non-diarrheic children were positive for Giardia, 8.0% (9/112) of diarrheic and 0.9% (1/113) of non-diarrheic children were positive for rotavirus (*p* < 0.01). Similarly, 2.7% (3/112) of diarrheic and 1.8% (2/113) of non-diarrheic children were positive for *Shigella* and 0.9% (1/112) of diarrheic and 0.9% (1/113) of non-diarrheic were positive for *Salmonella* (*p* > 0.05) (Table 3 and Figure 2).

**Table 3:** infection agents among children under five years of age in Ambagiorgis and Gedebge

Stool results and pathogens	Diarrheic (n = 112), (%)	Non-diarrheic (n = 113), (%)	Overall (n = 225), (%)	X^2^	P value
Negative	77 (68.8)	99 (87.6)	176 (78.2)	11.75	0.001
Positive	35 (31.3)	14 (12.4)	49 (21.8)		
***Giardia***					
No	89 (79.5)	104 (92.0)	193 (85.8)	7.29	0.007
Yes	23 (20.5)	9 (8.0)	32 (14.2)		
**Rotavirus**					
No	103 (92.0)	112 (99.1)	215 (95.6)	6.77	0.009
Yes	9 (8.0)	1 (0.9)	10 (4.4)		
***Shigella***					
No	109 (97.3)	111 (98.2)	220 (97.8)	0.21	0.644
Yes	3 (2.7)	2 (1.8)	5 (2.2)		
**Salmonella**					
No	111 (99.1)	112 (99.1)	223 (99.1)	0.001	0.995
Yes	1 (0.9)	1 (0.9)	2 (0.9)		

### Antibiotic susceptibility testing

For the five *Shigella* isolates obtained in the current study, susceptibility to seven antibiotics was recorded using the number of isolates and percentages calculated from five stool specimens. All five *Shigella* isolates screened for antibiotic susceptibility were resistant to ampicillin and amoxicillin and susceptible to ceftriaxone (Table 4). *Salmonella* isolates were ampicillin, amoxicillin- and tetracycline-resistant but were susceptible to chloramphenicol, gentamicin and ceftriaxone.

**Table 4:** Susceptibility of *Shigella* isolates to antibiotics in children under five years of age in Ambagiorgis and Gedebge

Antibiotics	*Shigella* isolates (n = 5)	
	Susceptible Number (%)	Resistant Number (%)
Tetracycline	2 (40.0)	3 (60.0)
Ampicillin	0 (0.0)	5 (100.0)
Chloramphenicol	3 (60.0)	2 (40.0)
Amoxicillin	0 (0.0)	5 (100.0)
Cotrimoxazol	3 (60.0)	2 (40.0)
Ceftriaxone	5 (100)	0 (0.0)
Gentamycin	2 (40.0)	3 (60.0)

## Discussion

In this study, 31% of diarrheic and 12% of non-diarrheic children were found to be positive for at least one infectious agent. Among the participating children, 65.3% were positive for *Giardia*, 20.4% for rotavirus, 10.2% for *Shigella* and 4.1% for *Salmonella*. Bloody stool, fever, vomiting, breastfeeding for children beyond 23 months of age; unsafe water source, wide-mouthed water storage containers, improper refuse disposal and lack of latrine facilities were more common among diarrheic than non-diarrheic children. Among non-vaccinated children, rotavirus infection was significantly higher in diarrheic than non-diarrheic children. The virus was not detected among children vaccinated against rotavirus, indicating a protective effect of the vaccine. *Giardia, Shigella* and *Salmonella* were not associated with the vaccination status of the children. In South Africa, up to 20,000 hospitalizations of children were prevented in the two years after the introduction of the vaccine [29]. The higher prevalence of rotavirus among non-vaccinated children coupled with the absence of positive cases among vaccinated children in the current study demonstrates the importance of the recently launched rotavirus vaccination program in controlling rotavirus in Africa.

In line with this study, the incidence of rotavirus gastroenteritis in Amarah City, Iraq [30] declined from 36% in unvaccinated children to 22% after they were vaccinated. In a study in Sudan [31], no significant difference was found in rotavirus prevalence between vaccinated (20%) and non-vaccinated (22%) children. These results may be linked to rotavirus serotypes that might not be included in the vaccine. Another possible explanation is that less than optimum vaccine handling techniques might have affected the efficacy of the vaccine [31]. In South Africa, rotavirus prevalence declined from 46% in 2009 to 29% in 2011 after the introduction of the vaccine [29]. In the current study, the 8.0% (9/112) prevalence of rotavirus is significantly smaller than the previously reported prevalence in Ethiopia, which ranged from 18% to 44% [20,32] and in other African countries, which has been reported as high as 46% [33]. The lower prevalence may be due to several factors, including improvements made to the different intervention programs, including implementation of rotavirus vaccination. In this study, the prevalence of *Giardia* was significantly higher among diarrheic than non-diarrheic children. A study conducted in Jimma Town in southwestern Ethiopia [34] reported a *Giardia* prevalence of 18% (8/44) among under-five children. Various global and national studies reported the prevalence of *Giardia* ranging from 9% to 42% [35,36]. The high *Giardia* and rotavirus infection rates in Wegera District appear to be due to poor sanitation, poor hygiene and inadequate water supplies [37] .

The prevalence of *Shigella* in the current study was lower (2.7% versus 4.6%) and the prevalence of *Salmonella* was higher (1.8% versus 1.1%) in our study area than in a previous study among patients with diarrhea in Gondar Town in northern Ethiopia [38]. A higher prevalence of *Shigella* was reported from Harar Town (7.0%) and from West Shoa Zone (11.0%), as was a higher prevalence of *Salmonella* reported from Harar (11.5%) and West Shoa Zone (9.0%) [39,40]. Bloody stools, vomiting and fever were observed more in diarrheic than non-diarrheic children. Fever and vomiting were the most common symptoms associated with diarrhea, corroborated by a study in Nigeria [41]. However, fever was not associated with the presence of acute diarrhea in children in Addis Ababa [42]. This difference might be due to errors that may have occurred during observation and measurement or linked to study methods and techniques used [43]. Frequent fever was detected in children with rotavirus, *Giardia, Shigella and Salmonella*. Fever is a good indication of whether a child is infected with viral, bacterial or other pathogens. In a similar study in Vellore, India, fever was significantly associated with child diarrhea episodes [44].

Environmental variables, including the source of household water supplies, type of water storage containers, refuse disposal methods and type of latrine facility, were significantly associated with acute childhood diarrhea in the current study. More households with diarrheic children used spring water than those in the non-diarrheic group. Spring water is commonly contaminated and is a vehicle for many microbial infections [7-12]. In line with this finding, unsafe water sources, including springs, were significantly associated with the occurrence of diarrheal disease in Jimma Town [45]. In the current study, improper refuse disposal was also more strongly associated with infectious agents isolated in diarrheic than non-diarrheic children. There was no proper waste management in the two study communities, resulting in widespread open defecation, which facilitates the transmission of diarrheal infectious agents. Households of diarrheic children in southern Ethiopia had fewer latrines than households of non-diarrheic groups, and children from households without toilet facilities were more likely to develop diarrhea [46]. Similarly, improper domestic waste disposal was significantly associated with the incidence of diarrhea in Ghana [47].

Although antibiotic susceptibility was carried out with a small number of *Shigella* isolates in the current study, which might limit the strength of the findings, the isolates were sensitive to ceftriaxone but were completely resistant to ampicillin and amoxicillin. This indicates a high level of antimicrobial resistance in the area, in agreement with a study in southern Ethiopia in which *Shigella* was completely resistant to ampicillin and amoxicillin [40]. This study has the following limitations: the cross-sectional study design used in this study might not allow us to make inferences regarding the causes of the diseases. Moreover, the study was limited to the wet season and mothers’/caretakers’ knowledge of previous diarrhea history was not included in the interviews. The findings are not completely free from recall bias of mothers/caregivers because the time reference for diarrhea was a two-week recall period. Another limitation of the study is that identification of the pathogens was not at the species level.

## Conclusion

This study revealed high prevalence of both enteropathogens and antimicrobial drug resistance in children under five years of age. Only ceftriaxone was highly effective against *Shigella* and *Salmonella* isolates. These two pathogens seem to have developed complete resistance against ampicillin and amoxicillin, implying that ceftriaxone should be considered when necessary within the context of use, considering that shigellosis is usually self-limiting in immune-competent children. Higher frequencies of bloody stool, vomiting and fever were observed among diarrheic than non-diarrheic children. Unsafe sources of water, wide-mouthed water storage containers, improper refuse disposal and lack of latrine facilities were more widespread in households of diarrheic than non-diarrheic children. We therefore recommend that rotavirus vaccination, hygienic practices, improved sanitation and household water supplies, and further study on the prevalence and drug resistance of bacterial pathogens be promoted in the study area.

### What is known about this topic

- There is a high prevalence of acute diarrhea among children under five years of age in Ethiopia;
- Rotavirus vaccination coverage for young children in Ethiopia is below 50%.

### What this study adds

- The prevalence of rotavirus infection was significantly higher among non-vaccinated than vaccinated diarrheic children. This information can inform local health departments about the need for the expansion of the rotavirus vaccination program for under-five children in the two study communities;
- The identification of Giardia enteropathogens in stool specimens of children under five years of age points out the need for promotion and advocacy of hygiene and behavioral intervention measures.

## Competing interests

The authors declare no competing interests.
